# Air Conditioning in Nursing Homes and Mortality During Extreme Heat

**DOI:** 10.1001/jamainternmed.2025.6595

**Published:** 2025-12-15

**Authors:** Gabrielle M. Katz, Kevin A. Brown, Vasily Giannakeas, Nathan M. Stall

**Affiliations:** 1 Temerty Faculty of Medicine, University of Toronto, Toronto, Ontario, Canada; 2 Public Health Ontario, Toronto, Ontario, Canada; 3 Dalla Lana School of Public Health, University of Toronto, Toronto, Ontario, Canada; 4 Women’s College Research Institute, Women’s College Hospital, Toronto, Ontario, Canada; 5 Division of General Internal Medicine and Geriatrics, Sinai Health and the University Health Network, Toronto, Ontario, Canada

## Abstract

**Importance:**

Extreme heat poses a serious health risk to older adults because they are generally more susceptible to heat-related mortality. Many nursing homes in the US and elsewhere lack air conditioning (AC). Ecological studies have evaluated the protective role of AC against mortality on extreme heat days in prisons and large urban settings; however, the nursing home setting remains understudied.

**Objective:**

To assess mortality rates during extreme heat days in nursing homes with AC compared to those without AC.

**Design, Setting, and Participants:**

This was a case-crossover study conducted in Ontario, Canada, to determine the odds of mortality associated with extreme days separated for nursing homes with and without AC using conditional logistic regression. Nursing home residents who died during the warm months (June to September) from 2010 to 2023 were included. Data were analyzed from June 2024 to April 2025.

**Exposures:**

An extreme heat day was defined as any day in the ≥90th percentile of the heat index (ambient temperature and relative humidity) for any given nursing home location during the study period.

**Main Outcomes and Measures:**

All-cause mortality during extreme heat days, by AC status of nursing homes.

**Results:**

Of the 73 578 deaths of nursing home residents from 2010 to 2023, 40 255 residents (mean [SD] age, 86.8 [8.8] years; 65.6% women) died at 276 homes with AC and 33 323 residents (mean [SD] age, 87.2 [8.7] years; 64.8% women) at 339 homes without AC. Before the AC mandate was announced in July 2020, nursing homes without AC (55.1%) were predominantly investor owned (ie, for profit; standardized mean difference [SMD], 0.47), were built to older design standards (SMD, 0.57), and had more residents per room (SMD, 0.58) compared with nursing homes with AC. Overall, 4889 deaths (13.8%) in nursing homes without AC occurred on extreme heat days compared with 4611 deaths (12.1%) in those with AC. Extreme heat was associated with significantly increased odds of mortality in nursing homes without AC (odds ratio [OR], 1.11; 95% CI, 1.06-1.16) but not in those with AC (OR, 1.03; 95% CI, 0.98-1.07). Compared to nursing homes with AC, those without AC were associated with significantly higher relative odds of mortality on extreme heat days (relative OR, 1.08; 95% CI, 1.01-1.15). Lagged analyses suggest that the associated effects of extreme heat persisted for 3 days beyond the initial exposure.

**Conclusion and Relevance:**

In this case-crossover study, mortality was lower during extreme heat days in nursing homes with AC compared to those without AC. These findings suggest that AC provision in nursing homes and other congregate care settings may be important for preventing mortality among older adults during extreme heat days.

## Key Points

**Question:**

What is the mortality associated with extreme heat that would be averted by requiring all nursing homes to provide air conditioning (AC)?

**Findings:**

This case-crossover study of 73 578 resident deaths in 2010 to 2023 found that extreme heat was associated with significantly increased odds of mortality in nursing homes without AC compared to those with AC (odds ratio, 1.11 vs 1.03, respectively). Overall, AC was associated with significantly lower relative odds of mortality on extreme heat days (relative odds ratio, 0.93).

**Meaning:**

These findings suggest that AC provision in nursing homes and other congregate care settings is important for preventing heat-related mortality.

## Teaser

This case-crossover study assesses mortality rates during extreme heat days in nursing homes with air conditioning compared to those without air conditioning.

## Introduction

Extreme heat poses a serious health risk to older adults (age ≥65 years).^1,2,3,4^ Globally, heat-related mortality among older adults reached a record high in 2023, increasing by 167% compared with the 1990s.^5^ In the US, heat-related deaths increased 117% from 1999 to 2023, and older adults accounted for 39% of heat-related deaths.^4,6^ Numerous factors contribute to heightened risk among older adults, including age-related physiological changes, use of certain prescription medications, and increased prevalence of chronic disease, functional impairment, and social isolation.^7,8,9,10,11^

Since the 1980s, the average number of heat waves per year in the US has nearly doubled, suggesting that heat-related mortality among older adults will continue rising.^12^ Air conditioning (AC) is recognized as an intervention to mitigate heat-related illness.^13,14,15,16,17,18^ In the US, there are more than 35 million people living in households without AC, including 7% of households in regions with above average temperatures.^19^ One area that has received considerably less attention is AC in nursing homes. Nursing home residents are vulnerable to extreme heat—many have limited mobility, multimorbidity, polypharmacy, and live with dementia.^20,21^

Under US federal law, nursing homes initially certified after October 1, 1990, must maintain a temperature of 71 to 81 °F (21.7-27.2 °C), yet according to data from the Centers for Medicare & Medicaid Services, 6898 of 14 782 active US nursing homes (46.7%) were certified before this date.^22^ In Ontario, Canada, there are more than 600 nursing homes with more than 76 000 residents, and in 2020, 55.1% of all nursing homes lacked AC serving residents’ rooms.^23^ At that time, homes without AC serving residents’ rooms were required to provide at least 1 designated cooling area served by AC for every 40 residents.^24^ This posed several challenges given that most nursing home residents live with cognitive impairment, many residents are bedbound, and during outbreaks of infectious diseases, including COVID-19, residents are isolated in their rooms. In July 2020, the provincial government of Ontario announced that all nursing homes must provide AC in resident rooms; the mandate officially took effect in June 2022.^24^ By May 2023, nearly all nursing homes (99.5%) had AC installed at a reported public cost of CAD$200 million (US$147.5 million in May 2023).^25^

Although previous ecological studies have provided evidence of the protective role of AC against mortality on extreme heat days in prisons and large urban settings,^13,14,15,16,17^ the nursing home setting is understudied. This case-crossover study evaluates mortality during extreme heat days in nursing homes with AC compared to those without AC.

## Methods

The study was approved by the Research Ethics Board of Mount Sinai Hospital (Toronto, Canada). The board waived the need for informed consent because data were deidentified and there was no contact with nursing home residents. We followed the Strengthening the Reporting of Observational Studies in Epidemiology (STROBE) reporting guideline.

### Study Design and Data Sources

We conducted a case-crossover analysis to investigate short-term mortality risk associated with extreme heat in nursing homes, stratified by AC status.^26,27,28,29^ The study was conducted across 615 licensed nursing homes in Ontario, Canada (16 million residents), from 2010 to 2023 during the warm months (June to September). In Ontario, all residents of nursing homes receive personal and nursing care as well as subsidized accommodation under a publicly funded program. Ontario nursing homes are not used as postacute care facilities and instead provide ongoing and 24-hour long-term care.

Data on nursing home residents and facility characteristics were obtained from the Ontario Ministries of Health and Long-Term Care. These data included sociodemographic and clinical characteristics from the validated Resident Assessment Instrument–Minimum Data Set, version 2.0 (RAI-MDS).^30,31^ The RAI-MDS is completed for all residents on admission to a nursing home, quarterly, and after any substantial health change.^32^ We included the most recent RAI-MDS assessment for each resident (closest to the date of death). We also used other provincial databases to capture resident demographic and facility characteristics (Continuing Care Reporting System [Canadian Institute for Health Information (CIHI)], Registered Persons Database [Ontario Ministry of Health], Client Profile Database [Ontario Ministry of Health], and Postal Code Conversion File Plus, version 8A1 [Statistics Canada]), physician services (National Ambulatory Care Reporting System [CIHI]), and hospital and emergency department use (Discharge Abstract Database [CIHI]). Data on facility characteristics included exact geographic location and dates of AC installation. Hourly temperature and humidity data for the geographic location of each nursing home was obtained from the open-source North American Land Data Assimilation System, which uses 0.125-degree spatial resolution (approximately a 12 × 12 km grid).^33^ We classified nursing homes as newer and older based on the Ontario Ministry of Long-Term Care’s definitions.^34,35^

### Case and Control Identification

The study population included all Ontario nursing home residents who died of any cause from 2010 to 2023 in June through September. The study used a self-controlled design that inherently eliminates time-invariant confounding. Characteristics such as resident sex, race, or geographic location do not differ between an individual’s date of death and self-matched control day, and therefore, cannot bias the exposure-outcome association.^26^

Case days were defined as the date when nursing home residents died. If case patients died within 14 days of a hospital admission, the case day was defined as the date of hospital admission. Deaths occurring more than 14 days after hospital admission were excluded. The control day was defined as 14 days prior to the case day (eFigure 1 in Supplement 1). A 14-day interval was selected to capture the acute nature of the association between extreme heat and mortality, while minimizing confounding from acute health deteriorations among nursing home residents unrelated to heat exposure. This interval also controls for confounding by seasonality and day of the week.

To be included, residents who died in nursing homes must have continuously resided in the same nursing home for at least 28 consecutive days leading up to their case day. Residents were excluded if their nursing home closed during the study period or if there were missing data on resident or home characteristics.

### Exposure Definition and Measurement

Extreme heat was measured by calculating the daily heat index for each nursing home using the algorithm from the 2011 US National Weather Service Calculator.^36^ The heat index accounts for both air temperature and relative humidity, which were measured for each nursing home location using data from the National Land Data Assimilation System. An extreme heat day was defined as any day with a heat index in ≥90th percentile compared to heat indexes measured in a given nursing home’s location across the entire study period.^13^ For nursing home residents who died in hospital, extreme heat exposure was recorded using their last date in the nursing home prior to death.

By considering several lag periods, we aimed to capture both acute and more prolonged effects of extreme heat exposure on mortality.^37,38,39^ We examined 3 lag periods (lag 0-1, lag 0-3, and lag 0-6, where lag 0 is the case day; eg, lag 0-1 is the date of death and 1 day prior, and so forth) Additional details are available in eFigure 1 in Supplement 1.

### Statistical Analysis

Statistical analyses were performed from June 2024 to April 2025 using SAS, version 9.4 (SAS Institute) and Python, version 3.14 (Python Software Foundation). We calculated descriptive statistics for the sociodemographic (age and sex; race and ethnicity were not available) and clinical characteristics of residents as well as nursing home characteristics, by AC status. AC status was determined by whether the nursing home had AC during the 28-day period prior to and including a resident’s death. We calculated standardized mean differences (SMDs) for sociodemographic and clinical characteristics of residents, with 10% (0.1) considered a meaningful difference.^40^

We calculated running averages (mean) of heat index over the lag periods and identified days that met the definition for extreme heat exposure. To assess the association between extreme heat exposure and mortality, we fit 2 conditional logistic regression models separately by AC status of nursing homes, with resident identifier as the strata variable demarcating the matched case-control pairs, and extreme heat as the only covariate. This meant that resident-level covariates that were invariant within strata were implicitly controlled for and could not be further adjusted. Odds ratios (ORs), 95% CIs, and *P* values were calculated for each lag period. All *P* values were calculated using 2-sided testing, and *P* <.05 was considered statistically significant. To capture the differential effects of extreme heat between nursing homes with and without AC, presented as relative ORs (RORs), a third conditional logistic regression was fitted to all nursing homes, and included extreme heat, AC status, and the interaction between heat and AC status as covariates. To examine potential effect modification across subgroups, we conducted analyses stratified by nursing home resident demographic and facility characteristics. To assess the robustness of the findings, we conducted a sensitivity analysis restricted to nursing home residents who died in their nursing home.

We also estimated deaths averted by analyzing the 336 homes that installed AC after the AC mandate was announced in July 2020. Using logistic regression, we modeled counterfactual mortality assuming no AC installation in these homes and compared predicted deaths to observed deaths. To estimate preventable deaths, had the Ontario AC mandate been implemented in 2010, we applied the same logistic regression approach to the 336 homes prior to AC installation, modeling mortality under a simulated scenario where AC had been present since 2010.

## Results

The analysis included 73 578 Ontario nursing home residents who died between 2010 and 2023 during June through September (Figure). Of these, 59 340 deaths (80.6%) occurred in nursing homes, and 14 238 (19.4%) occurred outside of nursing homes. Of the cohort, 50 209 residents (68.3%) were age 85 years or older; 25 629 were men (34.8%) and 47 949 were women (65.2%); and more than 70% were living with dementia (Table 1). There were 40 255 deaths in nursing homes with AC and 33 323 deaths in nursing homes without AC. SMDs were less than 0.1 for all sociodemographic and clinical characteristics of residents residing in homes with and without AC.

**Figure.**
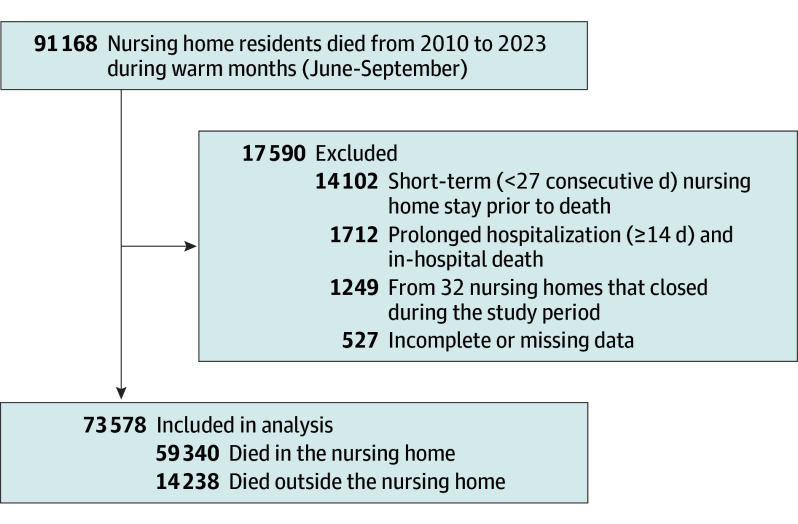
Flow Diagram of Study Participants

**Table 1.** Characteristics of Nursing Home Residents by Air Conditioning (AC), per Most Recent RAI-MDS^a^ Assessment

Characteristic	Residents, No. (%)		SMD
	Nursing home without AC	Nursing home with AC	
Residents, No.	33 323	40 255	NA
Age, y			
Mean (SD)	86.8 (8.8)	87.2 (8.7)	0.056
20-64	771 (2.3)	793 (2.0)	NA
65-69	778 (2.3)	817 (2.0)	NA
70-74	1401 (4.2)	1644 (4.1)	NA
75-79	2739 (8.2)	3215 (8.0)	NA
80-84	5223 (15.7)	5988 (14.9)	NA
85-89	8261 (24.8)	9745 (24.2)	NA
≥90	14 150 (42.5)	18 053 (44.8)	NA
Sex			
Female	21 865 (65.6)	26 084 (64.8)	.017
Male	11 458 (34.4)	14 171 (35.2)	
Comorbidities			
Diabetes	9614 (28.9)	11 748 (29.2)	0.007
Thyroid disease	7587 (22.8)	9225 (22.9)	0.004
Arteriosclerotic heart disease	7076 (21.2)	8295 (20.6)	0.016
Cardiac dysrhythmias	3942 (11.8)	4667 (11.6)	0.007
Hypertension	22 817 (68.5)	27 927 (69.4)	0.020
Chronic heart failure	6676 (20.0)	7806 (19.4)	0.016
Other cardiovascular diseases	9450 (28.4)	10 828 (26.9)	0.033
Arthritis	16 946 (50.9)	19 764 (49.1)	0.035
Osteoporosis	10 899 (32.7)	13 621 (33.8)	0.024
Dementia	23 716 (71.2)	29 036 (72.1)	0.021
Parkinson	2516 (7.6)	3044 (7.6)	0
Transient ischemic attack	2602 (7.8)	3127 (7.8)	0.002
Any psychiatric diagnosis	14 022 (42.1)	17 090 (42.5)	0.008
Asthma	1668 (5.0)	2074 (5.2)	0.007
Emphysema	6682 (20.6)	7457 (18.5)	0.052
Kidney failure	5389 (16.2)	6436 (16.0)	0.005
Cognitive performance scale score			
Mean (SD)	3.37 (1.68)	3.47 (1.68)	0.058
0	2153 (6.5)	2311 (5.7)	NA
1-2	6299 (18.9)	7281 (18.1)	NA
3-4	15 531 (46.6)	18 498 (46.0)	NA
5-6	9340 (28.0)	12 165 (30.2)	NA
ADL self-performance hierarchy scale score			
Mean (SD)	4.35 (1.24)	4.42 (1.24)	0.055
0	367 (1.1)	382 (0.9)	NA
1-2	1810 (5.4)	2103 (5.2)	NA
3-4	14 412 (43.2)	16 557 (41.1)	NA
5-6	16 734 (50.2)	21 213 (52.7)	NA
Bed-bound status	2563 (7.7)	3203 (8.0)	0.010
Feeding difficulties	457 (1.4)	555 (1.4)	0.001

Abbreviations: ADL, activities of daily living; NA, not applicable; SMD, standardized mean difference.

^a^ Resident Assessment Instrument–Minimum Data Set, version 2.0.^30,31^ Assessment data were collected for each resident from the date closest to inclusion as a case.

### Nursing Home Characteristics

As of July 2020, when the AC mandate for Ontario nursing homes was announced, there were 339 nursing homes without AC (55.1%) and 276 with AC (44.9%) (Table 2). Compared to nursing homes with AC, those without AC were situated in communities with higher income quintiles (SMD, 0.38), were predominantly for-profit (SMD, 0.47), built to older design standards (SMD, 0.57), had fewer beds (SMD, 0.24), and were more crowded (more residents per room and bathroom) (SMD, 0.58).

**Table 2.** Facility Characteristics of Nursing Homes, by Air Conditioning (AC) Status as of July 2020^a^

Characteristic	Nursing homes, No. (%)		SMD
	Without AC	With AC	
Nursing homes, No.	339	276	NA
Residential environment			
Rural	78 (23)	56 (20.3)	0.066
Urban	261 (77.0)	220 (79.7)	
Income quintile			
Mean (SD)	2.96 (1.38)	2.44 (1.35)	0.380
1	70 (20.6)	94 (34.1)	NA
2	62 (18.3)	63 (22.8)	NA
3	75 (22.1)	47 (17.0)	NA
4	75 (22.1)	47 (17.0)	NA
5	57 (16.8)	25 (9.1)	NA
Profit status			
For-profit	249 (73.5)	96 (34.8)	0.470
Not-for-profit	28 (8.3)	73 (26.4)	0.844
Municipal	62 (18.3)	107 (38.8)	0.505
Facility design standard^b^			0.565
Older	204 (60.2)	91 (33.0)	NA
Newer	135 (39.8)	185 (67.0)	NA
No. of beds			
Mean (SD)	121.7 (64.0)	139.4 (84.6)	0.239
≤64	72 (21.2)	60 (21.7)	NA
65-99	68 (20.1)	35 (12.7)	NA
100-199	165 (48.7)	130 (47.1)	NA
≥200	34 (10.0)	51 (18.5)	NA
Crowding index, mean (SD)	2.33 (0.75)	1.91 (0.68)	0.584

Abbreviations: NA, not applicable; SMD, standardized mean difference.

^a^ The intention for an AC mandate in Ontario nursing homes was publicly announced in 2020.

^b^ Newer facilities were defined as having at least 50% of beds meeting current design standards (meeting the 1999 or more recent standards), and older facilities were defined as having at least 50% of beds not meeting current design standards (meeting or falling below the 1972 design standards).

### Extreme Heat Experienced by Ontario Nursing Home Residents

Of 2233 case days, 878 (39.3%) met the definition of extreme heat, ranging from 38 days in 2023 (24.1%) to 85 days in 2021 (53.8%). From 2010 to 2023, there was a mean (range) of 63 (42-82) days per year when at least 1 nursing home experienced extreme heat (Table 3). The mean (range) number of extreme heat days per year experienced by a nursing home was 14 (1-63) days (eTable 1 in Supplement 1). Across all extreme heat days from June to September, the mean (range) heat index was 34.2 (28.3-49.8) °C. Across all calendar days from June to September, the mean (range) heat index was 26.2 (0 to 49.8) °C.

**Table 3.** Summary Measures of Extreme Heat in Nursing Homes Across Ontario, Canada, 2010 to 2023^a^

Year	Extreme heat days in ≥1 nursing home location, No.^b^	Extreme heat days only		All days from June to September	
		Mean heat index	Mean temperature, °C	Mean heat index	Mean temperature, °C
2010	59	34.6	31.5	26.3	25.2
2011	57	38.0	31.6	26.3	25.0
2012	69	36.6	31.5	25.4	24.3
2013	45	35.7	31.5	24.6	23.7
2014	42	33.6	31.5	24.4	23.8
2015	60	33.1	32.1	26.3	25.9
2016	82	32.9	32.2	28.4	28.1
2017	58	32.2	31.3	25.5	25.2
2018	81	33.6	32.3	28.4	27.7
2019	64	33.3	31.7	26.1	25.7
2020	73	33.0	32.3	27.1	26.7
2021	80	34.1	32.0	27.2	26.3
2022	56	34.1	31.4	26.4	25.7
2023	52	34.7	31.7	25.0	24.5

^a^ An extreme heat day was defined as any day ≥90th percentile of heat index (incorporates ambient temperature and relative humidity) for a respective nursing home location during the study period relative to other nursing homes included in the analysis.

^b^ Heat index was measured for each nursing home location, and extreme heat was defined with a percentile definition described; thus, not all nursing homes met criteria for extreme heat on the same calendar days.

### Outcomes

Overall, 4889 resident deaths (13.8%) occurred on extreme heat days in nursing homes without AC compared with 4611 deaths (12.1%) in homes with AC. Extreme heat was significantly associated with mortality (OR, 1.11; 95% CI, 1.06-1.16) in nursing homes without AC. Extreme heat was not significantly associated with mortality (OR, 1.03; 95% CI, 0.98-1.07) in nursing homes without AC (Table 4). Compared to nursing homes with AC, those without AC were associated with significantly higher relative odds of mortality on extreme heat days (ROR, 1.08; 95% CI, 1.01-1.15). The association was significant across lag periods 0 to 1 day (ROR, 1.08; 95% CI, 1.01-1.15) and 0 to 3 days (ROR, 1.08; 95% CI, 1.01-1.16), but not 0 to 6 days (ROR, 1.05; 95% CI, 0.98-1.12).

**Table 4.** Association of Air Conditioning (AC) With Mortality During Extreme Heat Exposure (EHE) Days by Nursing Home Air Conditioning Status, 2010 to 2023^a^

Lag day^b^	Nursing homes without AC (n = 276)			Nursing homes with AC (n = 339)			Relative odds of death on EHE days in nursing homes without AC (95% CI), d	*P* value
	No. (%)		Odds of death on EHE days, OR (95% CI), d	No. (%)		Odds of death on EHE days, OR (95% CI), d		
	Case days with EHE^c^^,^^d^	Control days with EHE^e^		Case days with EHE^f^	Control days with EHE			
0	4611 (13.8)	4245 (12.7)	1.11 (1.06-1.16)	4889 (12.2)	4752 (11.9)	1.03 (0.98-1.07)	1.08 (1.01-1.15)	.02
0-1	4152 (12.5)	3847 (11.5)	1.10 (1.05-1.15)	4312 (10.7)	4225 (10.6)	1.02 (0.97-1.06)	1.08 (1.01-1.15)	.02
0-3	4016 (12.1)	3711 (11.1)	1.10 (1.05-1.15)	4170 (10.4)	4088 (10.2)	1.02 (0.97-1.06)	1.08 (1.01-1.16)	.02
0-6	3822 (11.5)	3612 (10.8)	1.07 (1.02-1.13)	4006 (10.0)	3909 (9.8)	1.02 (0.98-1.07)	1.05 (0.99-1.12)	.15

Abbreviations: OR, odds ratio; ROR, relative odds ratio.

^a^ AC status of nursing homes was measured according to whether the nursing home had AC during the 28-day period prior to and including a resident’s death.

^b^ Lag 0 represents the case day and primary analysis, lag 0-1 represents the case day and 1 day prior, and so forth. For each lag period, a running mean of heat index was calculated, and only lag periods that met the definition for extreme heat are included in Table 4.

^c^ Case days are days in which a nursing home resident died during the study period or last day in the nursing home before a hospitalization where a nursing home resident died.

^d^ There was a total of 33 323 resident deaths during the study period in nursing homes without AC.

^e^ Control days are defined as 14 days prior to each case day.

^f^ There were a total of 40 255 resident deaths during the study period in nursing home with AC.

In analyses stratified by the nursing home residents’ demographic and facility characteristics, the association between a lack of AC and increased mortality on extreme heat days was consistent across nearly all subgroups (eTable 2 in Supplement 1). Associations were observed among residents who were younger than 80 years (ROR, 1.22; 95% CI, 1.04-1.42) and 90 years or older (ROR, 1.15; 95% CI, 1.05-1.26), male, had arteriosclerotic heart disease (ROR, 1.15; 95% CI, 1.00-1.32), and those residing in nursing homes in the lowest-income quintile (ROR, 1.15; 95% CI, 1.02-1.30) and the highest-income quintile (ROR, 1.24; 95% CI, 1.03-1.49. Data on facility characteristics were not available for 16 facilities, comprising 1052 residents (1.4%). A small number of strata yielded relative odds ratios less than 1, but these results were not statistically significant. In a sensitivity analysis restricted to residents who died within their nursing home (59 340 residents [80.6%]), results were consistent with the main analysis (eTable 3 in Supplement 1).

### Simulated Averted and Preventable Deaths

Ontario’s AC mandate was associated with 33 fewer nursing home resident deaths on extreme heat days (observed deaths, 308; simulated deaths if AC was not installed, 341) among 336 nursing homes that installed AC after the mandate (July 2020). This corresponds to a mean reduction of approximately 10 nursing home resident deaths per year from 2020 to 2023. If the AC mandate had been implemented in 2010, it would have been associated with 131 fewer deaths on extreme heat days (observed deaths, 4501; simulated deaths if AC had been present, 4370) in those 336 homes. This corresponds to a mean reduction of approximately 13 nursing home resident deaths per year from 2010 to 2020.

## Discussion

In this case-crossover study of all Ontario nursing home residents who died during the warm months of June to September from 2010 to 2023, we found that mortality was significantly higher during extreme heat days in nursing homes without AC compared to those with AC. Lagged analyses suggest that the effects of extreme heat persisted 3 days beyond initial exposure. We simulated that Ontario’s AC mandate averted 33 nursing home resident deaths, and 131 additional deaths could have been prevented had the mandate come into effect in 2010.

Our findings are consistent with epidemiologic studies reporting reductions in mortality during extreme heat days with the use of AC in other settings. Sera et al^14^ conducted a longitudinal study from 1972 to 2009 in 311 locations across Canada, Japan, Spain, and the US, and found that use of AC was independently associated with significant declines in heat-related mortality). Skarha et al^13^ reported that provision of AC in Texas prisons reduced mortality during extreme heat days in prisons with AC compared to those without. Our study adds to prior evidence of excess morbidity and mortality among nursing home residents during extreme heat and natural disasters when power outages interrupt electricity, and therefore, AC.^20,41,42,43,44^ Unlike community-dwelling older adults, most residents of nursing homes are dependent on facility infrastructure and protective government legislation to mitigate heat exposure.

The prevalence of AC in US nursing homes is not well described. The US Code of Federal Regulations requires that nursing homes initially certified after October 1, 1990, maintain a temperature range of 21.7 to 27.2 °C, but the means for maintaining this range (ie, AC) are not specified.^45^ Currently, 6898 of 14 782 active US nursing homes (46.7%) were certified before October 1, 1990; however, further information on the specific prevalence of AC in US nursing homes is unavailable.^22^ In our study, 204 Ontario nursing homes (60.2%) with older design standards did not install AC prior to the mandate.

Given the growing frequency, intensity, and duration of extreme heat events due to climate change, our findings underscore the need for universal access to AC in nursing homes. Importantly, the Ontario AC mandate requires provision of cooling in both common areas and resident rooms. The health effects of regular short-term exposure to common rooms or designated cooling centers with AC are not well studied. A laboratory-based study that simulated heat waves in older adults showed that a 2-hour exposure to AC during prolonged heat only temporarily reduces core body temperature and cardiovascular strain.^46^ Moreover, provision of AC in common areas alone is unlikely to fully protect residents who are bedbound, live with cognitive impairment, or are isolated due to infectious illnesses. Furthermore, the results of our study may be applied to other congregate care settings that house older adults, such as assisted living facilities and group homes.

In addition to AC, other measures can help mitigate the harms of extreme heat for nursing home residents. Clinical interventions include adjustments to prescription medications that can impair thermoregulation, such as diuretics, laxatives, anticholinergics, and antipsychotics.^47^ Physicians can also support residents with preventive measures, such as maintaining hydration, wearing lighter clothing, and staying indoors. Health systems can support nursing homes by requiring extreme heat action plans and by raising awareness of heat illness prevention. Where AC is unavailable, increasing shade with window shutters and/or trees, and combining fan use with skin wetting, can help reduce heat exposure.^48,49^ The use of fans, skin wetting, or both can reduce heat-induced cardiac strain among older adults at temperatures up to 38 °C; however, current CDC guidance for older adults advises against relying on fans as the sole cooling source during extreme heat.^50,51^

It is important to recognize that AC has a substantial carbon footprint. Accordingly, there is a need for more sustainable nursing home construction, including building homes outside of heat vulnerable areas, investing in green infrastructure to increase tree cover, and using passive cooling technologies. However, these strategies cannot be immediately deployed for the 1.3 million current US nursing home residents, and the many more living across the world who require immediate access to AC.^52^

There are important feasibility and cost considerations of mandating AC in nursing homes. Installing AC in the 336 Ontario nursing homes came at an estimated cost of CAD$ 200 million (US$147.5 million in May 2023) or approximately CAD$ 595 000 (US$ 430 000) per nursing home,^25^ presumably with additional ongoing costs to maintain the AC units. We estimated that 33 deaths were prevented through AC installation in these 336 homes. To better estimate the cost-effectiveness of Ontario’s investment, further research is needed on whether AC is also associated with reduced resident hospitalizations and emergency department visits, and improved resident quality of life and staff working conditions. An economic analysis should be the focus of future study.

### Strengths and Limitations

A strength of this study is its large study population, 73 578 nursing home residents across 615 Ontario nursing homes who died from 2010 to 2023. In addition, the case-crossover study design eliminated time-invariant confounding.

This study has several limitations. Its results are constrained by the binary classification of extreme heat in the logistic regression models and simulations, which may limit nuanced dose-response analyses. We incorporated a predefined minimum nursing home stay duration and maximal hospital stay duration in our inclusion criteria to improve the accuracy of heat exposure classification. We also conducted a sensitivity analysis restricted to residents who died within their nursing home (80.6%), the findings of which were consistent with our main results. Nonetheless, some degree of exposure status misclassification may have persisted. Although there were no clinically meaningful differences in baseline demographic and health characteristics between residents of nursing homes with and without AC, we acknowledge that there is no reference standard for measuring differential vulnerability to heat. A systematic review^53^ of 52 models assessing heat vulnerability risk found that factors such as age, socioeconomic status, race, health conditions, and social isolation are frequently included. In our analysis, we incorporated many of these domain; however, we were unable to access race-based data because it not routinely collected in Ontario. Although we cannot entirely rule out differences in residents’ heat susceptibility between nursing homes with and without AC, the similarity in observed baseline characteristics across facilities makes it unlikely that any such unmeasured differences explain our results.

## Conclusions

In this case-crossover study, we found that nursing homes without AC had significantly increased resident mortality during extreme heat days compared to nursing homes with AC. Our findings suggest that universal AC mandates such as the one implemented in Ontario, Canada, may protect nursing home residents and others in congregate care settings from heat-related mortality.

## Notes

Supplement 1. eFigure. Schematic representation of the time-stratified case-crossover design eTable 1. Extreme heat days by year per nursing home eTable 2. Stratified analyses by resident demographic and facility characteristics eTable 3. Sensitivity analysis restricted to deaths that occurred within nursing homes

Supplement 2. Data Sharing Statement
