# Supplementary material for: Air Conditioning in Nursing Homes and Mortality During Extreme Heat
Source: JAMA Intern Med. 2025 Dec 15;186(2):243–51. doi: 10.1001/jamainternmed.2025.6595 (PMC12706679; doi:10.1001/jamainternmed.2025.6595)
Supplement: Supplement 1. — eFigure. Schematic representation of the time-stratified case-crossover design eTable 1. Extreme heat days by year per nursing home eTable 2. Stratified analyses by resident demographic and facility characteristics eTable 3. Sensitivity analysis restricted to deaths that occurred within nursing homes [file jamainternmed-e256595-s001.pdf]

Supplemental Online Content

Katz GM, Brown KA, Giannakeas V, Stall NM. Provision of air conditioning in nursing homes and mortality during extreme heat. *JAMA Intern Med.* Published online December 15, 2025. doi:10.1001/jamainternmed.2025.6595

eFigure. Schematic representation of the time-stratified case-crossover design

eTable 1. Extreme heat days by year per nursing home

eTable 2. Stratified analyses by resident demographic and facility characteristics

eTable 3. Sensitivity analysis restricted to deaths that occurred within nursing homes

This supplemental material has been provided by the authors to give readers additional information about their work.

© 2025 Katz GM, et al. *JAMA Intern Med*.

eFigure. Schematic representation of the time-stratified case-crossover design

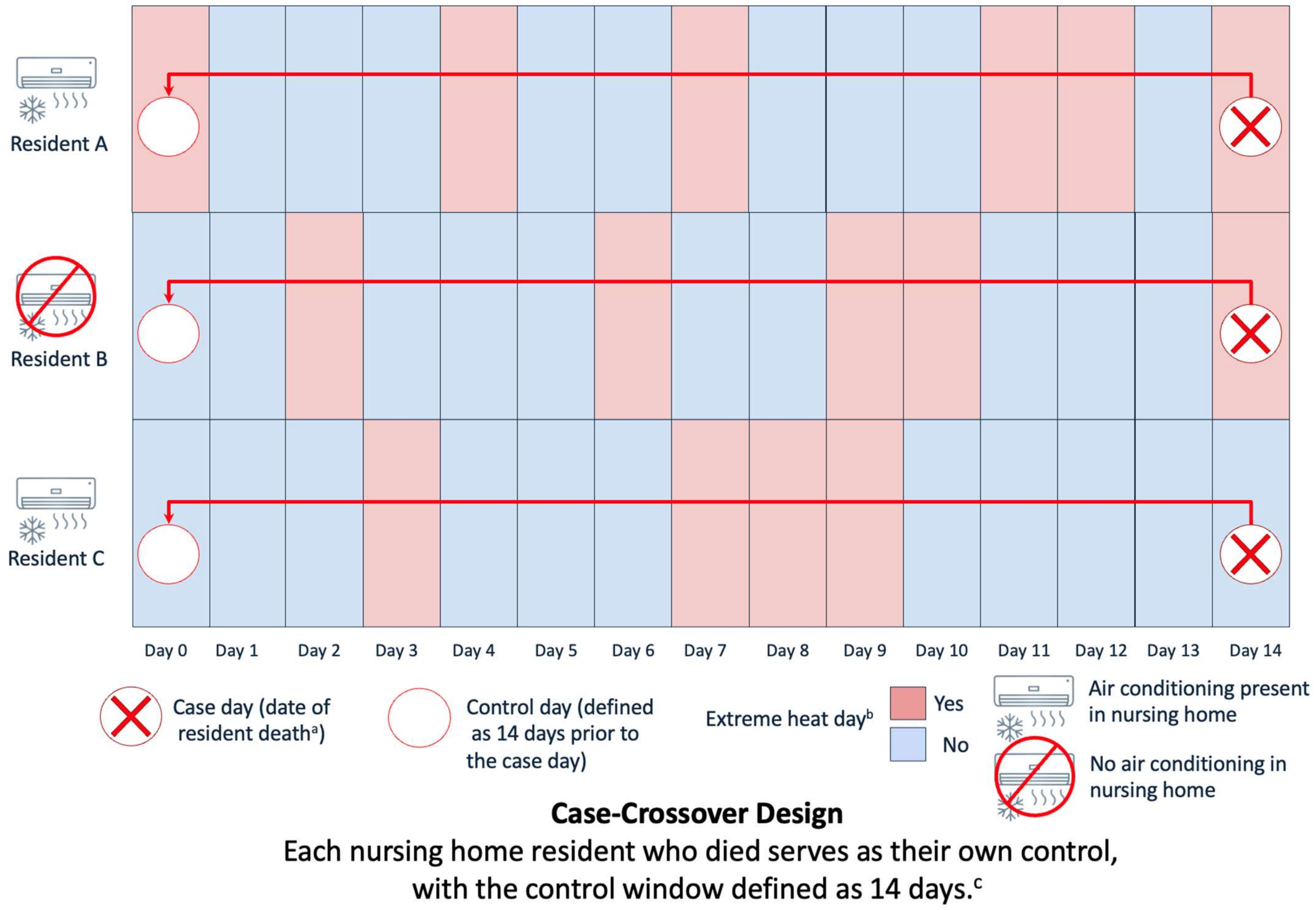

[a]If nursing home residents died in hospital, we assessed extreme heat exposure using the last date the resident was in their nursing home.

[b]Extreme heat days were defined as days that had ≥90th percentile of heat index.

[c]The study employed a self-controlled design that inherently eliminates time-invariant confounding. Stable characteristics such as sex, race, or geographic location do not differ between a resident's date of death and self-matched control day, and therefore cannot bias the exposure-outcome association.

© 2025 Katz GM, et al. *JAMA Intern Med.*

eTable 1. Extreme heat days by year per nursing home

Year	Average number of extreme heat days per facility	Minimum number of extreme heat days per facility	Maximum number of extreme heat days per facility
2010	12	1	39
2011	7	1	32
2012	5	1	52
2013	5	1	39
2014	4	1	38
2015	17	1	33
2016	33	1	63
2017	11	1	28
2018	33	1	58
2019	17	1	33
2020	24	1	42
2021	17	1	48
2022	11	1	33
2023	5	1	21

eTable 2. Stratified analyses by resident demographic and facility characteristics

Variable	Nursing Homes Without AC (n=276) OR (95% CI)			Nursing Homes With AC (n=339) OR (95% CI)				
	Case days with extreme heat exposure[b,c] n (%)	Control days with extreme heat exposure[d] n (%)	Odds of death on extreme heat days (95% CI)	Case days with extreme heat exposure[e] n (%)	Control days with extreme heat exposure n (%)	Odds of death on extreme heat days (95% CI)	Relative odds of death on extreme heat days in nursing homes without AC (95% CI)	P-value
Age <80 (years)	780 (2.3)	680 (2.0)	1.18 (1.06, 1.32)	742 (1.8)	758 (1.9)	0.97 (0.87, 1.08)	1.22 (1.04, 1.42)	0.01174
Age 80-89 (years)	1,843 (5.5)	1,787 (5.3)	1.04 (0.97, 1.12)	1,965 (4.9)	1,816 (4.5)	1.09 (1.02, 1.16)	0.96 (0.87, 1.06)	0.4057
Age 90+ (years)	1,988 (6)	1,778 (5.3)	1.14 (1.07, 1.23)	2,182 (5.4)	2,178 (5.4)	1.00 (0.94, 1.06)	1.15 (1.05, 1.26)	0.003817
Cognitive performance scale score (0-2)	1,107 (3.3)	1028 (3.1)	1.11 (1.01, 1.22)	1,145 (2.8)	1,147 (2.9)	1.01 (0.93, 1.10)	1.10 (0.97, 1.25)	0.1427
Cognitive performance scale score (3-4)	2,207 (6.6)	2,,071 (6.2)	1.11 (1.04, 1.18)	2,,318 (5.8)	2,259 (5.6)	1.05 (0.98, 1.11)	1.06 (0.97, 1.16)	0.2083
Cognitive performance scale score (5-6)	1,297 (3.9)	1,146 (3.4)	1.11 (1.01, 1.20)	1426 (3.5)	1,346 (3.4)	1.01 (0.93, 1.10)	1.09 (0.97, 1.23)	0.1363
ADL Self Performance Hierarchy scale score (0-2)	282 (0.8)	289 (0.9)	1.07 (0.90, 1.28)	284 (0.7)	310 (0.8)	1.00 (0.84, 1.19)	1.07 (0.84, 1.37)	0.5643
ADL Self Performance Hierarchy scale score (3-4)	2,033 (6.1)	1,947 (5.8)	1.12 (1.05, 1.20)	2,072 (5.1)	2,145 (5.4)	1.01 (0.95, 1.08)	1.11 (1.01, 1.22)	0.02597
ADL Self Performance Hierarchy scale score (5-6)	2,296 (6.9)	2,009 (6)	1.10 (1.03, 1.17)	2,533 (6.3)	2,297 (5.7)	1.05 (0.99, 1.11)	1.05 (0.96, 1.15)	0.2892
Crowding index (<2)[f]	2,041 (6.1)	1,927 (5.7)	1.08 (1.01, 1.15)	3,609 (9.0,)	3,545 (8.9)	1.02 (0.97, 1.07)	1.06 (0.98, 1.15)	0.1621
Crowding index (≥2)[f]	2,570 (7.7)	2,318 (6.9)	1.13 (1.07, 1.20)	1,280 (3.2)	1207 (3)	1.06 (0.97, 1.15)	1.07 (0.96, 1.19)	0.2032
Female sex	3,010 (9)	2,779 (8.3)	1.10 (1.04, 1.17)	3148 (7.8)	3,025 (7.6)	1.04 (0.99, 1.10)	1.06 (0.98, 1.15)	0.1319

© 2025 Katz GM, et al. *JAMA Intern Med.*

Male sex	1,601 (4.8)	1,466 (4.4)	1.12 (1.03, 1.20)	1,741 (4.3)	1,727 (4.3)	1.00 (0.93, 1.08)	1.11 (1.00, 1.23)	0.04597
Diabetes mellitus	1,344 (4)	1,237 (3.7)	1.09 (1.01, 1.19)	1,411 (3.5)	1,383 (3.5)	1.00 (0.93, 1.09)	1.09 (0.97, 1.22)	0.1439
Thyroid disease	1,026 (3.1)	1,008 (3)	1.01 (0.92, 1.11)	1,150 (2.9)	1,038 (2.6)	1.10 (1.01, 1.20)	0.92 (0.81, 1.05)	0.198
Arteriosclerotic heart disease	987 (3)	876 (2.6)	1.13 (1.02, 1.25)	1,007 (2.5)	1,003 (2.5)	0.98 (0.90, 1.08)	1.15 (1.00, 1.32)	0.04351
Cardiac dysrhythmias	521 (1.6)	510 (1.5)	1.01 (0.88, 1.15)	573 (1.4)	566 (1.4)	0.99 (0.88, 1.12)	1.02 (0.85, 1.22)	0.8684
Hypertension	3,228 (9.7)	2,930 (8.7)	1.11 (1.05, 1.17)	3,435 (8.5)	3,307 (8.3)	1.02 (0.97, 1.08)	1.08 (1.00, 1.16)	0.03904
Chronic heart failure	921 (2.8)	825 (2.5)	1.10 (1.00, 1.22)	975 (2.4)	894 (2.2)	1.07 (0.97, 1.18)	1.03 (0.90, 1.18)	0.6828
Other cardiovascular diseases	1,293 (3.9)	1,201 (3.6)	1.07 (0.98, 1.17)	1,363 (3.4)	1286 (3.2)	1.04 (0.96, 1.13)	1.03 (0.91, 1.16)	0.6499
Arthritis	2,365 (7.1)	2,201 (6.6)	1.08 (1.01, 1.14)	2,464 (6.1)	2,362 (5.9)	1.03 (0.97, 1.10)	1.04 (0.96, 1.14)	0.3436
Osteoporosis	1,523 (4.6)	1379 (4.1)	1.11 (1.03, 1.20)	1,673 (4.2)	1,584 (4.0)	1.05 (0.97, 1.13)	1.06 (0.95, 1.18)	0.2792
Dementia	3,327 (10)	3,,036 (9.1)	1.10 (1.05, 1.16)	3,567 (8.9)	3,387 (8.5)	1.04 (0.99, 1.09)	1.06 (0.99, 1.14)	0.1174
Parkinson’s	332 (1)	309 (0.9)	1.08 (0.91, 1.27)	387 (1.0)	357 (0.9)	1.08 (0.92, 1.26)	1.00 (0.80, 1.25)	0.9903
TIA	365 (1.1)	328 (1.0)	1.12 (0.95, 1.31)	379 (0.9)	383 (1.0)	0.97 (0.83, 1.13)	1.15 (0.92, 1.43)	0.2172
Any psychiatric diagnosis	1,899 (5.7)	1,759 (5.2)	1.08 (1.01, 1.16)	2,051 (5.1)	1,989 (5.0)	1.02 (0.95, 1.09)	1.06 (0.97, 1.17)	0.2048
Asthma	201 (0.6)	225 (0.7)	0.86 (0.70, 1.06)	246 (0.6)	236 (0.6)	1.03 (0.85, 1.25)	0.84 (0.63, 1.11)	0.2088
Emphysema	910 (2.7)	867 (2.6)	1.04 (0.94, 1.15)	880 (2.2)	886 (2.2)	0.97 (0.88, 1.07)	1.07 (0.93, 1.24)	0.3218
Renal failure	778 (2.3)	697 (2.1)	1.11 (1.00, 1.24)	756 (1.9)	749 (1.9)	0.98 (0.88, 1.09)	1.14 (0.97, 1.32)	0.1061
Bed bound status	357 (1.1)	252 (0.8)	1.20 (1.01, 1.42)	378 (0.9)	292 (0.7)	1.06 (0.90, 1.25)	1.13 (0.89, 1.43)	0.3203
Feeding difficulties	56 (0.2)	50 (0.1)	1.12 (0.75, 1.68)	64 (0.2)	55 (0.1)	1.12 (0.76, 1.64)	1.00 (0.57, 1.75)	0.9906

© 2025 Katz GM, et al. *JAMA Intern Med.*

Residential environment (Rural)	800 (2.4)	732 (2.2)	1.11 (1.00, 1.24)	623 (1.5)	612 (1.5)	1.02 (0.90, 1.14)	1.10 (0.93, 1.29)	0.261
Residential environment (Urban)	3,811 (11.4)	3,513 (10.5)	1.11 (1.05, 1.16)	4,,266 (10.6)	4,140 (10.3)	1.03 (0.98, 1.08)	1.08 (1.01, 1.15)	0.0329
Income quintile (1)	984 (3.0)	850 (2.5)	1.19 (1.08, 1.31)	1630 (4.0)	1,576 (3.9)	1.04 (0.96, 1.11)	1.15 (1.02, 1.30)	0.02492
Income quintile (2)	890 (2.7)	849 (2.5)	1.06 (0.96, 1.17)	1,122 (2.8)	1,117 (2.8)	1.00 (0.92, 1.09)	1.06 (0.93, 1.21)	0.3867
Income quintile (3)	982 (2.9)	903 (2.7)	1.11 (1.01, 1.22)	902 (2.2)	807 (2)	1.12 (1.02, 1.24)	0.99 (0.86, 1.14)	0.8661
Income quintile (4)	940 (2.8)	888 (2.6)	1.07 (0.97, 1.18)	871 (2.2)	852 (2.1)	1.02 (0.92, 1.13)	1.05 (0.91, 1.21)	0.4766
Income quintile (5)	815 (2.4)	755 (2.3)	1.10 (0.99, 1.22)	364 (0.9)	400 (1)	0.89 (0.76, 1.03)	1.24 (1.03, 1.49)	0.02325
Profit status (for-profit)	3,282 (9.8)	2,984 (8.9)	1.12 (1.07, 1.19)	1,631 (4.1)	1,,570 (3.9)	1.03 (0.96, 1.11)	1.09 (0.99, 1.19)	0.06437
Profit status (municipal)	515 (1.5)	503 (1.5)	1.04 (0.91, 1.18)	1,576 (3.9)	1,556 (3.9)	1.01 (0.94, 1.09)	1.02 (0.88, 1.19)	0.7752
Profit status (not-for-profit)	814 (2.4)	758 (2.3)	1.09 (0.98, 1.21)	1,682 (4.2)	1,626 (4.1)	1.04 (0.96, 1.12)	1.05 (0.93, 1.20)	0.4301
Number of beds (≤64)	498 (1.5)	467 (1.4)	1.08 (0.95, 1.24)	407 (1.0)	393 (1.0)	1.03 (0.89, 1.20)	1.05 (0.86, 1.28)	0.6507
Number of beds (65-99)	773 (2.3)	688 (2.1)	1.15 (1.03, 1.28)	449 (1.1)	434 (1.1)	1.03 (0.90, 1.19)	1.11 (0.93, 1.33)	0.2441
Number of beds (100-199)	2,524 (7.6)	2,339 (7)	1.10 (1.04, 1.17)	2,,483 (6.2)	2,390 (6)	1.04 (0.98, 1.10)	1.06 (0.97, 1.15)	0.1733
Number of beds (200+)	816 (2.4)	751 (2.2)	1.11 (1.00, 1.23)	1,550 (3.9)	1,535 (3.8)	1.01 (0.93, 1.09)	1.10 (0.97, 1.25)	0.15
Home design standard (newer home)	2,086 (6.3)	1,971 (5.9)	1.08 (1.01, 1.15)	3,587 (8.9)	3,529 (8.8)	1.01 (0.96, 1.07)	1.06 (0.98, 1.15)	0.1539
Home design standard (older home)	2,484 (7.5)	2,230 (6.7)	1.14 (1.07, 1.21)	1,266 (3.1)	1,194 (3.0)	1.06 (0.97, 1.15)	1.07 (0.97, 1.19)	0.1741

[a]Lag 0 represents the case day and primary analysis, lag 0-1 represents the case day and one day prior, and so forth. For each lag period, a running average of heat index was calculated, and only lag periods that met the definition for extreme heat are included in Table 4.
[b]Case days are days in which a nursing home resident died during the study period or last day in the nursing home before a hospitalization where a nursing home resident died.
[c]There were a total of 33,323 resident deaths during the study period in nursing homes without AC.

© 2025 Katz GM, et al. *JAMA Intern Med.*

[d]Control days are defined as 14 days prior to each case day.
[e]There were a total of 40,255 resident deaths during the study period in nursing home with AC.
[f]Crowding index refers to the mean number of occupants per room and bathroom across an entire nursing home according to the following equation: $N_{residents} \div (½N_{bedrooms} + ½N_{bathrooms})$

© 2025 Katz GM, et al. *JAMA Intern Med*.

eTable 3. Sensitivity analysis restricted to deaths that occurred within nursing homes (n = 59,340, 80.6% of all cases)

Lag Day[a]	Nursing Homes Without AC (n=276) OR (95% CI)			Nursing Homes With AC (n=339) OR (95% CI)				
	Case days with extreme heat exposure[b,c] n (%)	Control days with extreme heat exposure[d] n (%)	Odds of death on extreme heat days (95% CI)	Case days with extreme heat exposure[e] n (%)	Control days with extreme heat exposure n (%)	Odds of death on extreme heat days (95% CI)	Relative odds of death on extreme heat days in nursing homes without AC (95% CI)	P-value
0	3,760 (14.1)	3,472 (12.9)	1.10 (1.05, 1.16)	4,018 (12.3)	3,943 (12.15)	1.02 (0.97, 1.06)	0.92 (0.86, 0.98)	0.016
0-1	3,358 (12.6)	3,149 (11.7)	1.08 (1.03, 1.14)	3,522 (10.8)	3,512 (10.8)	1.00 (0.95, 1.05)	0.92 (0.86, 0.99)	0.023
0-3	3,231 (12.1)	3,048 (11.3)	1.08 (1.02, 1.13)	3,423 (10.5)	3,396 (10.5)	1.00 (0.95, 1.06)	0.93 (0.87, 1.00)	0.059
0-6	3,101 (11.6)	2,966 (11.0)	1.06 (1.00, 1.12)	3,278 (10.1)	3,273 (10.1)	1.00 (0.95, 1.05)	0.94 (0.87, 1.01)	0.104

AC, air conditioning; OR, odds ratio; CI, confidence interval; ROR, relative odds ratio

[a]Lag 0 represents the case day and primary analysis, lag 0-1 represents the case day and one day prior, and so forth. For each lag period, a running average of heat index was calculated, and only lag periods that met the definition for extreme heat are included in Table 4.

[b]Case days are days in which a nursing home resident died during the study period or last day in the nursing home before a hospitalization where a nursing home resident died.

[c]There were a total of 33,323 resident deaths during the study period in nursing homes without AC.

[d]Control days are defined as 14 days prior to each case day.

[e]There were a total of 40,255 resident deaths during the study period in nursing home with AC.

© 2025 Katz GM, et al. *JAMA Intern Med.*
