# Supplementary material for: Air Conditioning in Nursing Homes and Mortality During Extreme Heat
Source: JAMA Intern Med. 2025 Dec 15;186(2):243–51. doi: 10.1001/jamainternmed.2025.6595 (PMC12706679; doi:10.1001/jamainternmed.2025.6595)
Supplement: Supplement 2. — Data Sharing Statement [file jamainternmed-e256595-s002.pdf]

Data Sharing Statement

Katz. Air Conditioning in Nursing Homes and Mortality During Extreme Heat. *JAMA Intern Med*.
Published December 15, 2025. doi:10.1001/jamainternmed.2025.6595

Data
Data available: Yes
Data types: Data dictionary
How to access data: nathan.stall@sinaihealth.ca
When available: With publication

Supporting Documents
Document types: None

Additional Information
Who can access the data: Researchers whose proposed use of the data has been approved
Types of analyses: For a specified purpose.
Mechanisms of data availability: N/A (Data dictationary alone being made available).
Any additional restrictions: N/A.
